# Huaier Extract Induces Autophagic Cell Death by Inhibiting the mTOR/S6K Pathway in Breast Cancer Cells

**DOI:** 10.1371/journal.pone.0131771

**Published:** 2015-07-02

**Authors:** Xiaolong Wang, Wenwen Qi, Yaming Li, Ning Zhang, Lun Dong, Mingjuan Sun, Jinjing Cun, Yan Zhang, Shangge Lv, Qifeng Yang

**Affiliations:** 1 Department of Breast Surgery, Qilu Hospital, Shandong University, Jinan, Shandong, P.R. China; 2 School of Medicine, Shandong University, Jinan, Shandong, P.R. China; 3 Qilu Hospital, Shandong University, Jinan, Shandong, P.R. China; 4 Department of Pathology Tissue Bank, Qilu Hospital, Shandong University, Jinan, Shandong, P.R. China; School of Medicine, University of Belgrade, SERBIA

## Abstract

Huaier extract is attracting increased attention due to its biological activities, including antitumor, anti-parasite and immunomodulatory effects. Here, we investigated the role of autophagy in Huaier-induced cytotoxicity in MDA-MB-231, MDA-MB-468 and MCF7 breast cancer cells. Huaier treatment inhibited cell viability in all three cell lines and induced various large membranous vacuoles in the cytoplasm. In addition, electron microscopy, MDC staining, accumulated expression of autophagy markers and flow cytometry revealed that Huaier extract triggered autophagy. Inhibition of autophagy attenuated Huaier-induced cell death. Furthermore, Huaier extract inhibited the mammalian target of the rapamycin (mTOR)/S6K pathway in breast cancer cells. After implanting MDA-MB-231 cells subcutaneously into the right flank of BALB/c nu/nu mice, Huaier extract induced autophagy and effectively inhibited xenograft tumor growth. This study is the first to show that Huaier-induced cytotoxicity is partially mediated through autophagic cell death in breast cancer cells through suppression of the mTOR/S6K pathway.

## Introduction

Breast cancer is the predominant type of cancer among women and the leading cause of cancer-related mortality [1, 2]. Significant advances in treatment have improved patient survival rates and quality of life, but more successful treatments are still required [3, 4]. Indeed, some traditional methods, such as chemotherapy, may cause severe side effects and drug resistance in patients. Therefore, it is of utmost importance to explore new approaches for targeting breast cancer in order to reduce morbidity and mortality.

Natural dietary products have been widely and safely consumed over centuries, and preclinical studies suggest that some have potential applications in pharmacology and cancer therapy [5]. In recent years, Huaier extract has attracted increased attention due to its biological activities, including antitumor [6], anti-parasite [7] and immunomodulatory effects [8]. In our previous studies, we have shown that Huaier extract exerts a strong anti-proliferative effect by inducing caspase-dependent apoptosis, suppressing the estrogen receptor α pathway, and inhibiting angiogenesis in breast cancers [9–11]. However, it is still not known if Huaier extract triggers other forms of cell death such as autophagy.

Autophagy refers to an evolutionally conserved catabolic process in which a cell degrades long-lived proteins and damaged organelles, including the endoplasmic reticulum, the Golgi apparatus, and the mitochondria [12]. It is thought to be an essential long-term survival mechanism for when cells suffer nutrient starvation. Inhibition of autophagy results in a rapid cell death under conditions of starvation or during withdrawal of growth factors [13]. However, several studies have demonstrated that autophagy is not only a survival response, but also an important molecular mechanism for tumor cell suicide [14]. Recently, extensive studies have revealed autophagy to be a promising and potential new strategy for fighting human diseases, including cancer [15, 16]. Compared with the caspase-dependent apoptosis, autophagic cell death is dependent on the presence of autophagosomes and autolysosomes, presumably due to irreversible massive self-destruction of cellular contents or activation of death signal pathways [17]. In human breast cancer cells, some anticancer agents, such as acetonic extract of Buxus sempervirens [18], Eupatorium odoratum [19], or Sirtinol [20], have been demonstrated to induce autophagic cell death.

In this study, we investigated the anti-cancer effect of Huaier extract on MDA-MB-231, MDA-MB-468 and MCF7 human breast cancer cell lines both in vitro and in vivo. We found that Huaier extract inhibited growth of these cell types by inducing autophagic cell death and we examined the signal pathways involved in Huaier-induced autophagy. To the best of our knowledge, this is the first study to demonstrate that Huaier extract induces autophagic cell death through the mTOR/S6K pathway in human breast cancer cells. These results suggest that Huaier extract could be an attractive therapeutic adjuvant for the treatment of human breast cancers.

## Materials and Methods

### Cell culture and reagents

Huaier extract was kindly provided by Gaitianli Medicine Co., Ltd. (Jiangsu, China) and prepared as described in [9]. The human breast cancer cell lines MDA-MB-231, MDA-MB-468 and MCF7 were purchased from the American Type Culture Collection (ATCC, Manassas, VA, USA) and were routinely cultured in DMEM medium (Gibco-BRL, Rockville, IN, USA), containing 10% FBS (Haoyang Biological Manufacturer Co., Ltd., Tianjin, China), 100 U/ml penicillin and 100 μg/ml streptomycin. T47D cells were cultured in RPMI-1640 medium (Gibco-BRL) with 10% fetal bovine serum and 10 μg/ml bovine insulin (Sigma-Aldrich, St. Louis, MO, USA). All cells were maintained in a humidified atmosphere containing 5% CO_2_ at 37°C. Both 3-Methyladenine (3-MA), chloroquine (CQ), monodansylcadaverine (MDC) and acridine orange were obtained from Sigma-Aldrich.

### MTT assay

The MTT assay was performed in order to determine cell viability [21]. In brief, 2×10^3^ breast cancer cells per well were seeded in 96-well plates and allowed to attach overnight at 37°C. Culture medium containing vehicle or drugs was then added to the medium in each well and incubated for indicated time intervals. At indicated time points, the cells in the 96-well plate were incubated with 20 μl MTT in a growth medium. After 4–6 hours of incubation at 37°C, the supernatants were carefully aspirated and formazan crystals were solubilized with 100 μl dimethyl sulfoxide (DMSO). The absorbance values at 490 nm were determined with a Microplate Reader (Bio-Rad, Hercules, CA, USA). Data were presented as the percentage of survival rate relative to the vehicle-treated control.

### Acridine orange/ethidium bromide (AO/EB) double staining assay

AO/EB double staining was performed as previously described in [22]. Both control and treated cells were stained with 4 μg/ml acridine orange and 4 μg/ml ethidium bromide for 15 min. The stained cells were examined under the fluorescence microscope in both red and green channels. Viable cells have uniform bright green nuclei with normal structure, whereas early apoptotic cells have green nuclei, but their nuclei are more condensed. Late apoptotic cells have orange to red nuclei with condensed chromatin, whereas necrotic cells have orange to red nuclei with normal chromatin levels.

### Terminal Deoxynucleotidyl Transferase-mediated Nick End Labeling (TUNEL) assay

TUNEL staining was performed using the One Step TUNEL Apoptosis Assay Kit (Beyotime, Jiangsu, China) according to the manufacturer’s instructions. After treatment, the cells were fixed with 4% paraform phosphate buffer saline, rinsed with PBS, and then permeabilized with 0.1% Triton X-100 for 2 min on ice followed by the application of the TUNEL kit for 1 hour at 37°C. The TUNEL-positive cells (red fluorescence) were imaged using fluorescent microscopy.

### Transfection with small interfering RNA

The small interfering RNA (siCON and siLC3B) were purchased from Genepharma (Shanghai, China). Transfection was performed with lipofectamine 2000 (Invitrogen) according to the manufacturer’s protocol.

### Electron microscopy

In order to detect the induction of autophagy in Huaier-treated tumor cells, we performed an ultrastructural analysis under electron microscopy. In brief, the cells were treated with 4 mg/ml Huaier for 48h before being fixed with a solution containing 2.5% glutaraldehyde plus 2% paraformaldehyde in 0.1 M cacodylate buffer, pH 7.3, for 1 h. After fixation, the samples were post-fixed in 1% OsO_4_ in the same buffer for 1 h and analyzed under the electron microscope. Representative areas were chosen for ultrathin sectioning and viewed with a JEM 1010 transmission electron microscope (JEOL, Peabody, MA) at an accelerating voltage of 80 kV. Digital images were obtained with an AMT imaging system (Advanced Microscopy Techniques, Danvers, MA).

### Immunofluorescence staining

In order to study the formation of autophagic vacuoles, LC3B, a specific marker of autophagosomes, was used and detected by immunofluorescence staining. In brief, the cells were grown on coverslips in the 24-well plates for 3 days before being treated with 4 mg/ml Huaier extract for 48 h. After fixation with 4% paraformaldehyde for 25 min at room temperature, the cells were permeabilized with 0.1% Triton-X 100 in PBS (PBST) for 15 min. The cells were blocked with 10% goat serum in PBS, and incubated with rabbit anti-LC3B monoclonal antibody (Cell Signaling Technology) and rhodamine-conjugated anti-rabbit secondary antibody (Kirkegaard & Perry Laboratories, Inc.). Nuclear DNA was stained with 4, 6-diamidino-2-pheny-lindole (DAPI) for 10 min. Finally, anti-fading medium was added and the coverslips were immediately observed under a DP71 fluorescence microscope (Olympus, Tokyo, Japan).

### Monodansylcadaverine (MDC) staining

Autophagic vacuoles were detected with MDC by incubating the cells with MDC (50 μM) in PBS at 37°C for 20 min. After incubation, the cells were repeatedly washed (three times) with PBS and immediately analyzed by a DP71 fluorescence microscope (excitation wavelength, 380 nm; emission filter, 525 nm).

### Western blot analysis

Vehicle- or drug-treated cells were lysed in a lysis buffer containing 50 mM Tris-HCl, pH 7.5, 150 mM NaCl, 1% Nonidet P-40, 0.25% sodium deoxycholate, 0.1% SDS with protease inhibitors. Lysates were centrifuged at 12,000 rpm for 15 min. Supernatants were collected, subjected to electrophoresis on 12% (for LC3B antibody) or 10% (for other primary antibody) SDS-polyacrylamide gel and transferred to polyvinylidene fluoride membranes (ImmobilonP; Millipore, Bedford, MA, USA). The membrane was blocked with 5% non-fat dry milk for 1h before being incubated with the indicated primary antibody (Cell Signaling Technology) overnight at 4°C. The membrane was then treated with horseradish peroxidase conjugated secondary antibodies and the signals were detected by enhanced chemiluminescence.

### Flow cytometry analysis of acidic vesicular organelles (AVO)

In order to quantify the change in the number of AVO in cells treated with Huaier extract, indicated cells were stained with acridine orange (1 μg/ml) in PBS at 37°C for 15 min in darkness. The cells were washed with PBS twice and then suspended in PBS for immediate analysis. The data were analyzed using BD Cell Quest software.

### Ethics statement

Animal experiments were performed in strict accordance with the Guidelines for the Care and Use of Laboratory Animals of Shandong University. The protocol was approved by the Ethics Committee of Qilu Hospital, Shandong University (Permit Number: 12019). Animals were humanely CO_2_ euthanized and every effort was made to minimize suffering.

### In vivo tumorigenesis assay

The in vivo tumorigenesis assay was performed as previously described in [23]. MDA-MB-231 cells (2×10^6^) were injected subcutaneously into the right flank of 4-5-week-old BALB/c nu/nu female mice. After 2 days, the mice were randomly assigned to vehicle, Huaier alone and Huaier + CQ treatment groups. The Huaier group was given a 100 μl solution containing 50 mg Huaier extract by gavage, administered daily. Chloroquine (15 mg/kg) was injected intraperitoneally once daily. Tumor growth was measured every 4 days and tumor volume was calculated using the following equation:

$$volume=\left(width^{2}\times length\right)÷2$$

After 32 days, the mice were sacrificed and the xenografts were removed for immunohistochemical staining.

### Immunohistochemical analysis

Immunohistochemistry was performed as described in [24]. After excision, the tumor tissues were stored in 10% neutral-buffered formalin. After 24 h, the samples were paraffin-embedded and sliced into 4 μm sections. The sections were microwaved for antigenic retrieval and incubated with primary antibody overnight at 4°C. The sections were then washed with PBS, treated with biotinylated anti-immunoglobulin antibody for 20 min, and allowed to react with horseradish peroxidase-conjugated streptavidin. Following detection with diaminobenzidine, the sections were counterstained with hematoxylin. The representative images of tumor tissues were taken by an Olympus light microscope.

### Statistical analysis

The software SPSS (version 18.0) was used for statistical analysis. A student’s *t*-test and one-way ANOVA were performed to determine significance. All error bars represent the standard error (SEM) of three experiments and differences with p < 0.05 were considered significant.

## Results

### Huaier extract induced cell death and morphological changes in human breast cancer cell lines

**Fig 1:**
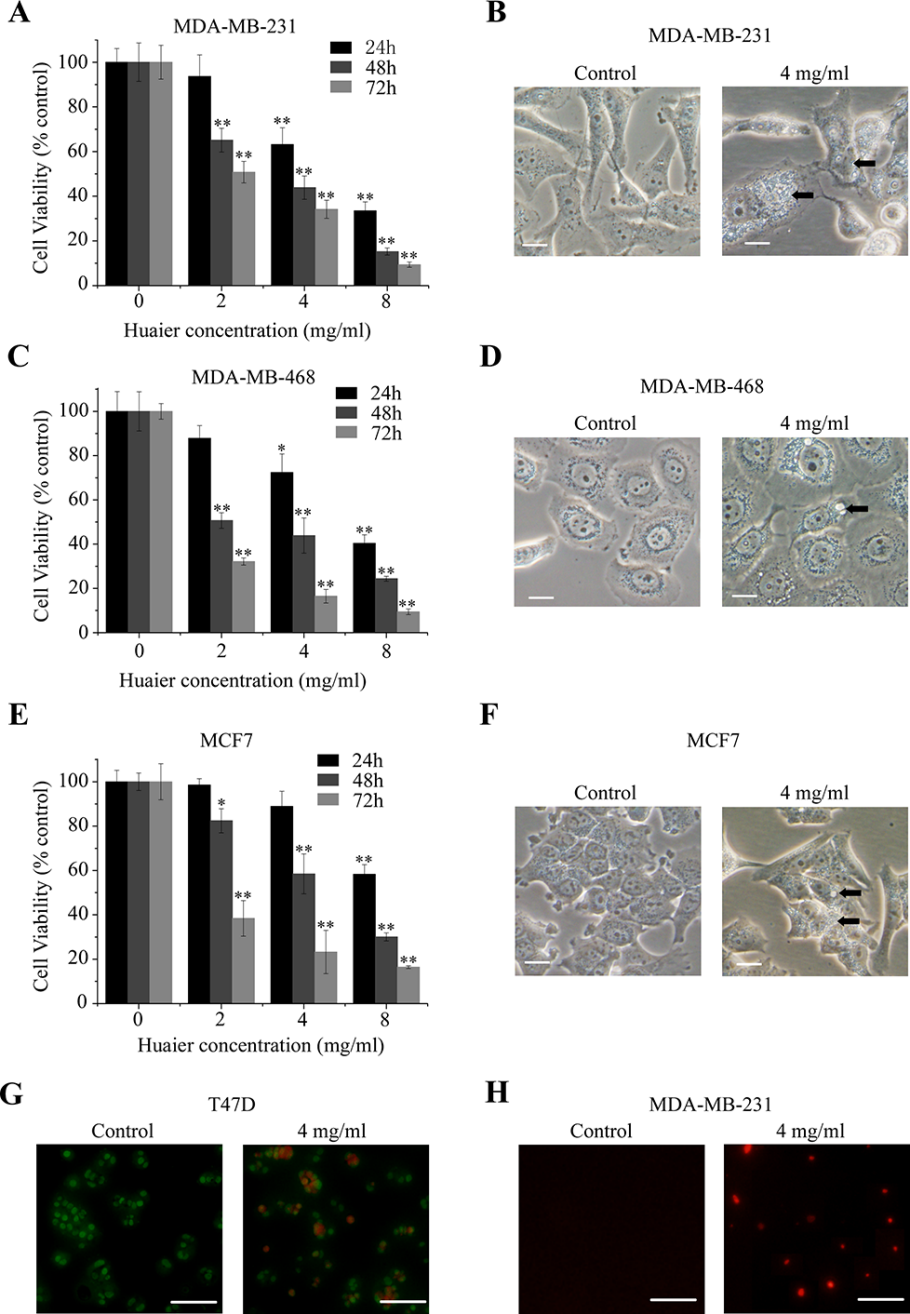
Huaier extract reduced cell viability and triggered intracellular vesicular organelles in breast cancer cells. (A, C and E) The cytotoxic effect of Huaier extract was measured using the MTT assay. Cells were treated with different concentrations of Huaier extract over indicated time periods and subjected to the MTT assay. The viability of untreated cells was considered 100%. The experiments were performed in triplicate and data is presented as the mean ± SD of three separate experiments. ^*^p < 0.05, ^**^p < 0.01. (B, D and F) Micrographs show the appearance of vesicular organelles in breast cancer cells after 48 h of exposure to Huaier extract. Arrows indicate the intracellular vacuoles. Bars, 10 μm. (G) AO/EB staining of T47D cells was performed to detect apoptosis and necrosis induced by Huaier extract. Bars, 50 μm. (H) Representative TUNEL staining (red fluorescence) of MDA-MB-231 cells treated with or without Huaier extract. Bars, 50 μm.

We examined the effect of Huaier extract on cell viability in MDA-MB-231, MDA-MB-468 and MCF7 cells. Huaier extract did indeed decrease cell viability in all three cancer cell lines in a dose- and time-dependent manner, as shown in Fig 1A, 1C and 1E). We observed that 4 mg/ml Huaier extract could significantly reduce cell viability with an incubation period of 48 h (56.16 ± 5.22% in MDA-MB-231; 56.13 ± 7.98% in MDA-MB-468; 41.54 ± 9.03% in MCF7). Furthermore, in Huaier-treated cells, we detected the appearance of large membranous vacuoles in the cytoplasm which is a characteristic feature of cells undergoing autophagy (Fig 1B, 1D and 1F). Therefore, we hypothesized that Huaier extract could induce autophagic cell death in human breast cancer cells.

We used AO/EB staining and the TUNEL assay to detect other modes of cell death induced by Huaier extract. As shown in Fig 1G and 1H, Huaier extract could also induce necrosis and apoptosis, which is consistent with our previous data [9].

### Huaier extract induced autophagy in breast cancer cells

**Fig 2:**
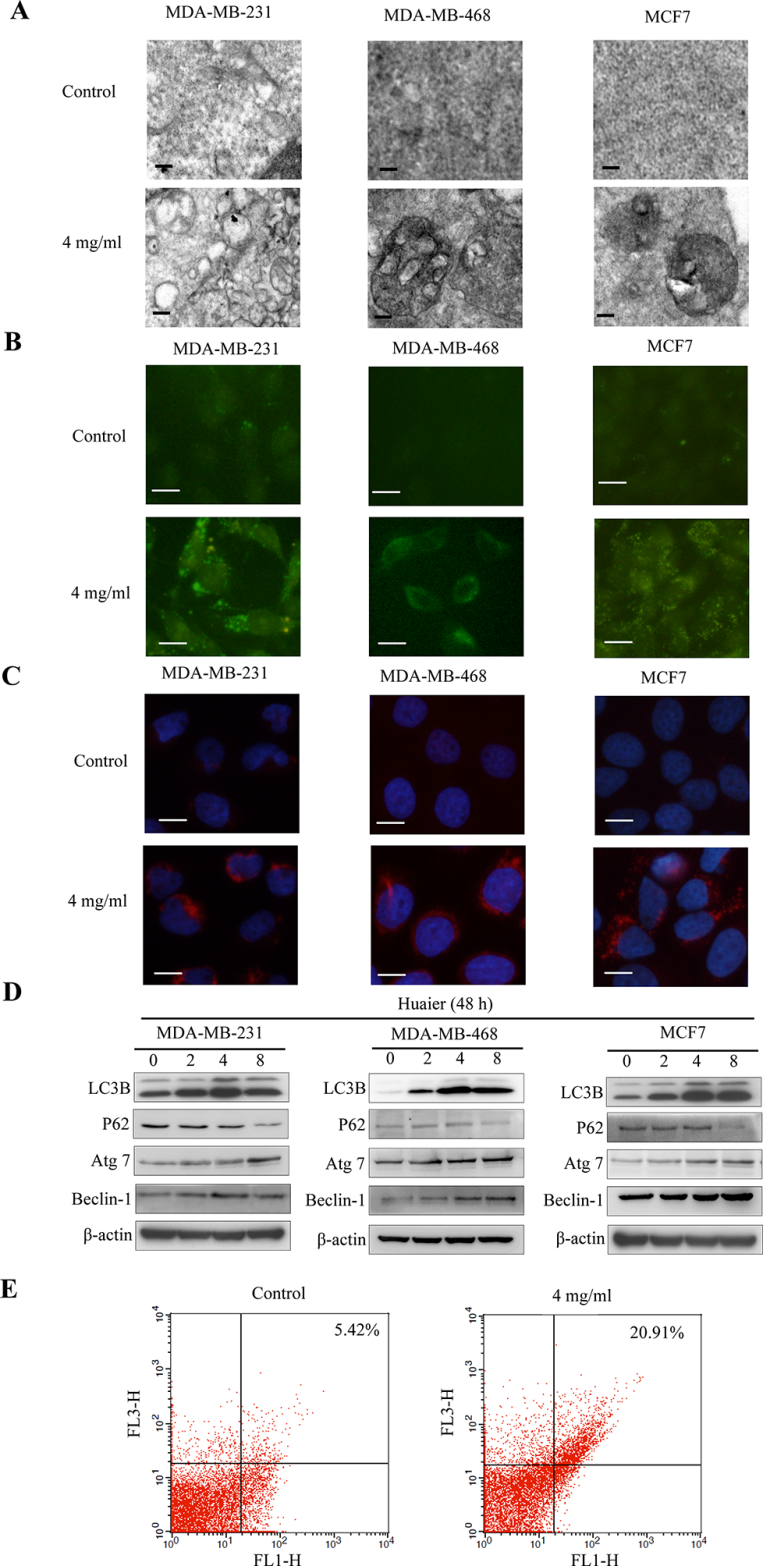
Huaier extract induced autophagy in breast cancer cells. (A) Representative electron micrographs of breast cancer cells treated with or without 4 mg/ml Huaier extract for 48 h. Bars, 100nm. (B) Acidic vesicular organelles induced by Huaier extract were stained with MDC. Bars, 10 μm. (C) Aggregation of LC3B in Huaier-treated cells. Cells were treated with or without 4 mg/ml Huaier extract and stained with the LC3B antibodies using immunofluorescence staining. Puncta represent the autophagosome formation. Bars, 10 μm. (D) Cell lysates were harvested after incubation with different concentrations of Huaier extract for 48h. β-actin was used as a loading control. (E) Cells in suspension were labeled with acridine orange and quantified using flow cytometry. FL1-H indicates green color intensity (cytoplasm and nucleus), whereas FL3-H shows red color intensity (AVO). Cells in up quadrants were considered AVO-positive. Results shown are representative of three independent experiments.

Stimulus-induced autophagy causes ultrastructural changes in the treated cells. Therefore, we tested whether autophagy occurred in Huaier-treated cells, which we assessed using transmission electron microscopy. As shown in Fig 2A, exposure of cells to 4 mg/ml Huaier extract for 48 h resulted in the appearance of autophagocytic vacuoles containing extensively degraded cellular material or membranous structures, consistent with the characteristics of cells undergoing autophagy.

Monodansylcadaverine (MDC) is a specific marker for autophagic vacuoles. Fig 2B shows that Huaier-treated cells exhibited higher fluorescent density and more MDC-labeled particles compared with vehicle-treated control cells, indicating that Huaier extract could increase MDC recruitment to autophagosomes in the cytoplasm of cells.

Microtubule-associated protein 1 light chain 3 (LC3) is the first known mammalian protein that is specifically associated with the autophagosomal membrane. During autophagy, cytosolic LC3-Ⅰ is cleaved to form the membrane-associated LC3-Ⅱ, which is involved in the formation of autophagosomes [25]. As shown in Fig 2C, the number and intensity of punctuate LC3B fluorescence increased after 48 h of 4 mg/ml Huaier extract treatment, indicating the induction of autophagy. We next investigated the expression of several autophagy-related genes using the immunoblot assay. The data revealed a significant increase of processed LC3B-Ⅱ, Atg7, Beclin-1 and a decrease of selective autophagy target p62/SQSTM1 in a dose-dependent way (Fig 2D).

Furthermore, the induction of autophagy by Huaier extract was confirmed by flow cytometry using acridine orange staining in order to detect acidic vesicular organelles (AVOs). As shown in Fig 2E, Huaier treatment resulted in increased formation of AVOs. These results suggested that autophagy was activated in response to Huaier extract.

### Huaier extract induced autophagy-associated cell death in breast cancer cells

**Fig 3:**
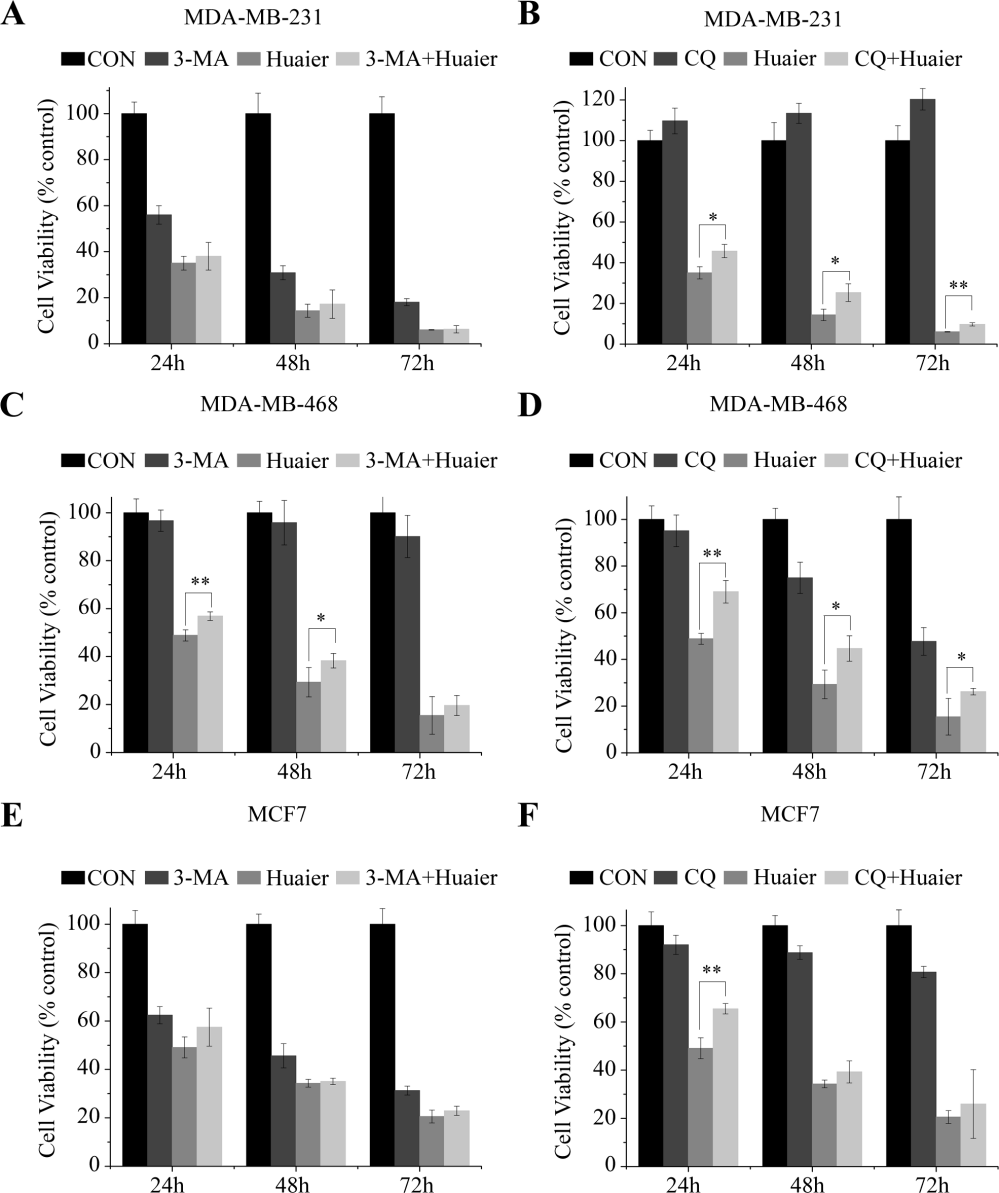
The effect of autophagy inhibition on the cytotoxicity of Huaier extract. MDA-MB-231 (A and B), MDA-MB-468 (C and D) and MCF7 (E and F) were pretreated with 3-MA or CQ before being exposed to Huaier extract for the indicated time. The cell viability was measured using the MTT assay. The experiments were performed in triplicate and data presented as the mean ± SD of three separate experiments. ^*^p < 0.05, ^**^p < 0.01.

Next, we used the autophagy inhibitors 3-methyladenine (3-MA) and chloroquine (CQ) to investigate whether the induction of autophagy contributed to Huaier-induced cell death. 3-MA is a well-known inhibitor of autophagosome formation, whereas CQ inhibits lysosome acidification and degradation [26]. The cytotoxic effect of Huaier extract combined with 3-MA and CQ treatment was measured by MTT. Pretreatment with 4 μM 3-MA resulted in rescued cell viability (Fig 3A, 3C and 3E). In addition, 20 μM CQ could significantly reduce the cell death in all three cell lines (Fig 3B, 3D and 3F).

**Fig 4:**
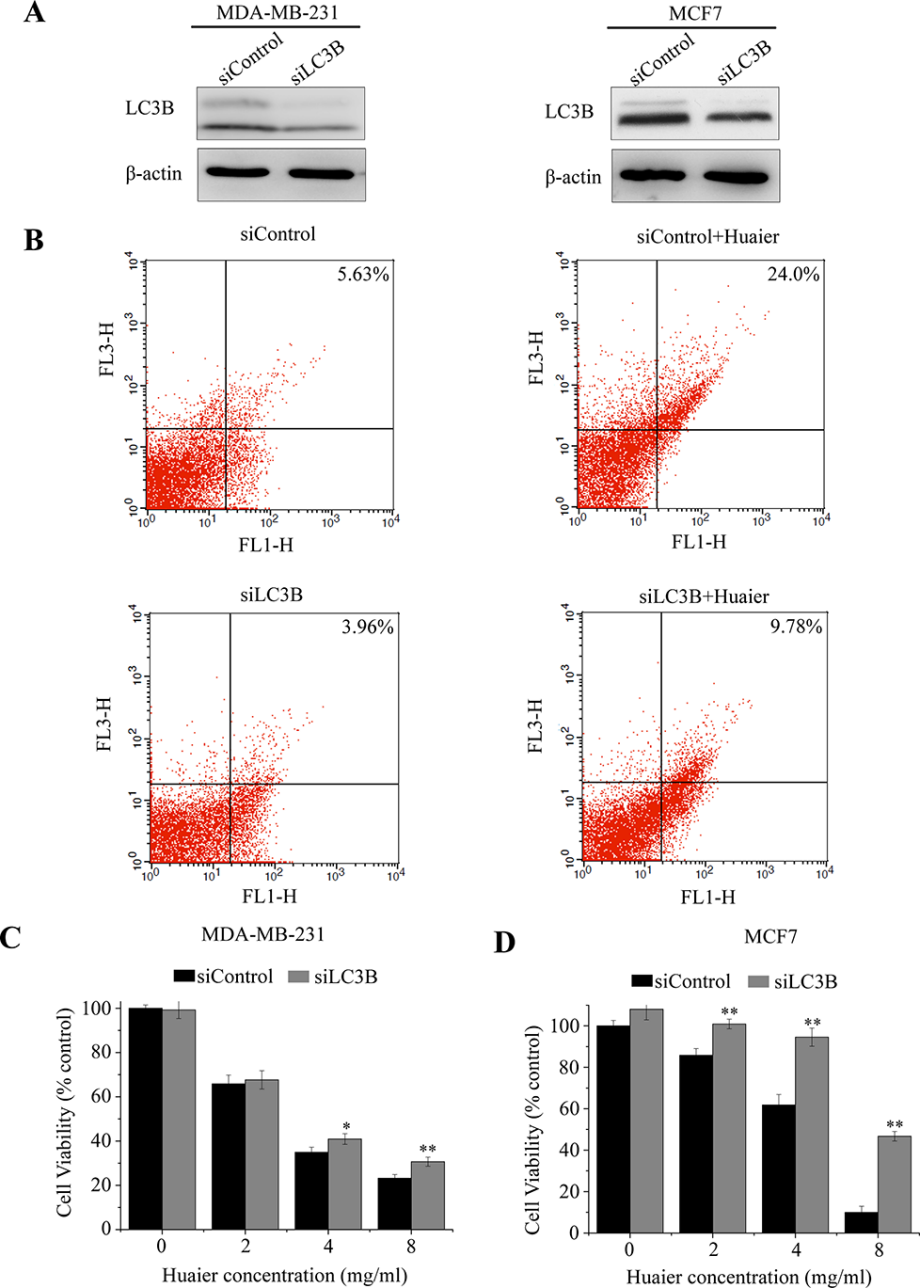
LC3B siRNA blocks Huaier-mediated autophagic cell death. (A) LC3B expression was knocking-down by siRNA. MDA-MB-231 and MCF7 cells were transfected with siLC3B. siControl was used as the negative control. (B) Huaier-mediated autophagy was blocked by LC3B siRNA. MDA-MB-231 cells were treated with siControl, siControl+Huaier, siLC3B, or siLC3B+Huaier. Autophagy was detected by flow cytometry. (C and D) The cell viability was measured using the MTT assay. The experiments were performed in triplicate and data presented as the mean ± SD of three separate experiments. ^*^p < 0.05, ^**^p < 0.01.

We then applied a genetic approach to confirm the effect of autophagy inhibition. We used siRNA to specifically knock down LC3B in MDA-MB-231 and MCF7 cells (Fig 4A). Using flow cytometry-AVO analysis, we observed that Huaier treatment enhanced autophagy induction, but transfection with siLC3B blocked the effect of Huaier extract (Fig 4B). As shown in Fig 4C and 4D, gene silencing with small interfering LC3B RNA (siLC3B) suppressed the cytotoxic effect of Huaier extract. These data suggested that inhibition of Huaier-induced autophagy recovered the reduced cell viability in MDA-MB-231, MDA-MB-468 and MCF7 cells.

### Huaier extract inhibited the activity of mTOR/S6K pathway

**Fig 5:**
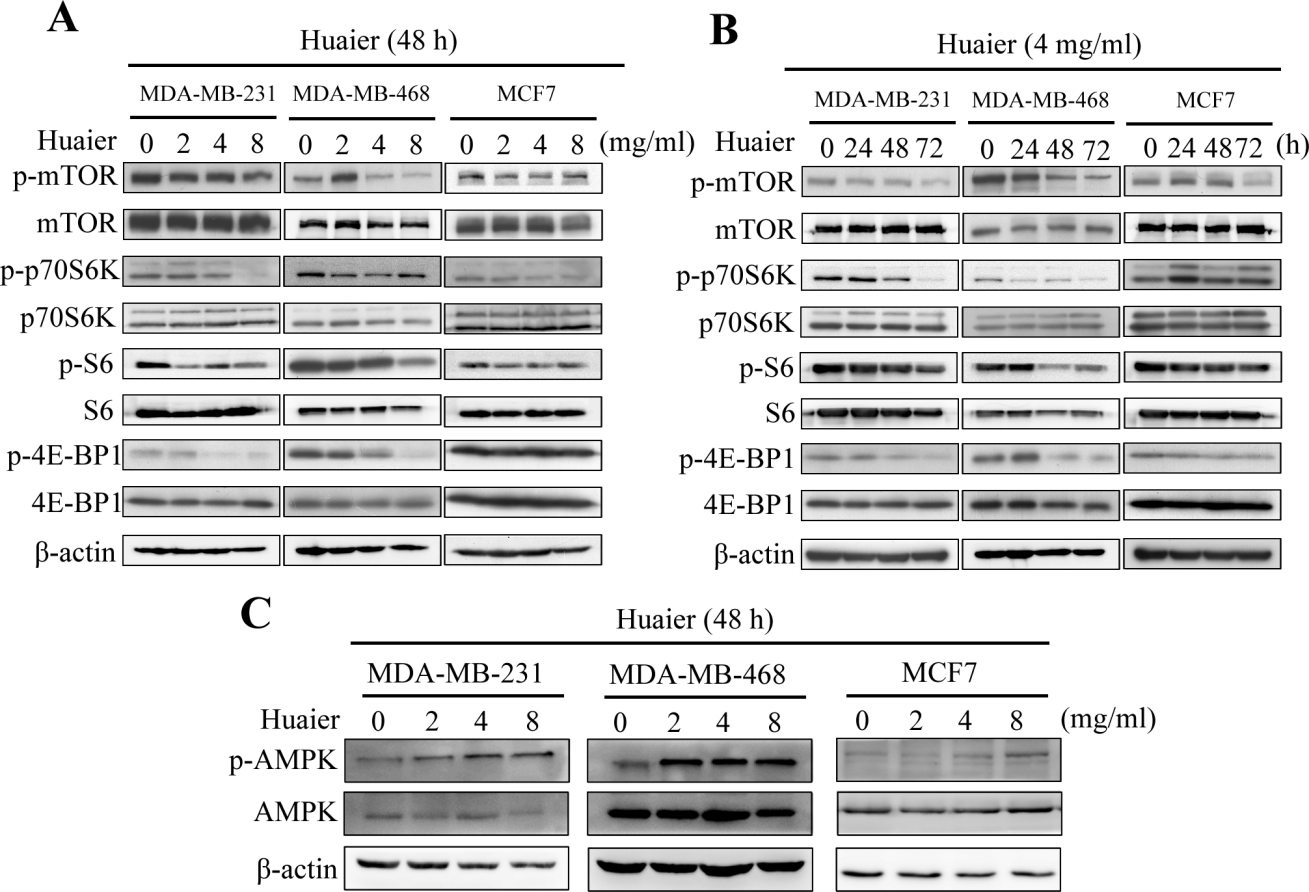
Huaier extract inhibited the activity of mTOR/S6K pathway. (A and B) Cells were treated with various concentrations of Huaier extract for the indicated time. Cell lysates were prepared and detected via Western blotting. The effect of Huaier extract on the levels of mTOR, p70S6K, S6, 4E-BP1 and their phosphorylated forms are shown. (C) AMPK was activated after Huaier treatment in a dose-dependent manner. Results are representative of three independent experiments.

Recent studies have indicated that inhibition of mTOR/S6K pathway is associated with the triggering of autophagy in cancer cells [27, 28]. Therefore, we investigated whether the mTOR/S6K pathway was involved in Huaier-induced autophagic cell death in all three breast cancer cell lines using Western blotting. As shown in Fig 5A and 5B, Huaier extract caused a decrease in levels of the phosphorylated form of mTOR, whilst total mTOR levels remained unaffected by the treatment. Furthermore, Huaier extract induced a sharp reduction in the phosphorylation of the mTOR downstream targets p70 ribosomal protein S6 kinase (p70S6K), S6 ribosomal protein (S6) and 4E-BP1, suggesting a potent inhibitory effect of Huaier extract on mTOR/S6K signaling.

We also explored the activation of AMP-activated protein kinase (AMPK) in Huaier treated breast cancer cell lines as AMPK is one of the principal mTOR inhibitors [29, 30], we also explored the activation of AMPK in Huaier treated breast cancer cell lines. As shown in Fig 5C, AMPK could be activated by Huaier extract in a dose-dependent manner in all three cell lines.

### Huaier extract inhibited growth of subcutaneous tumors by inducing autophagy

**Fig 6:**
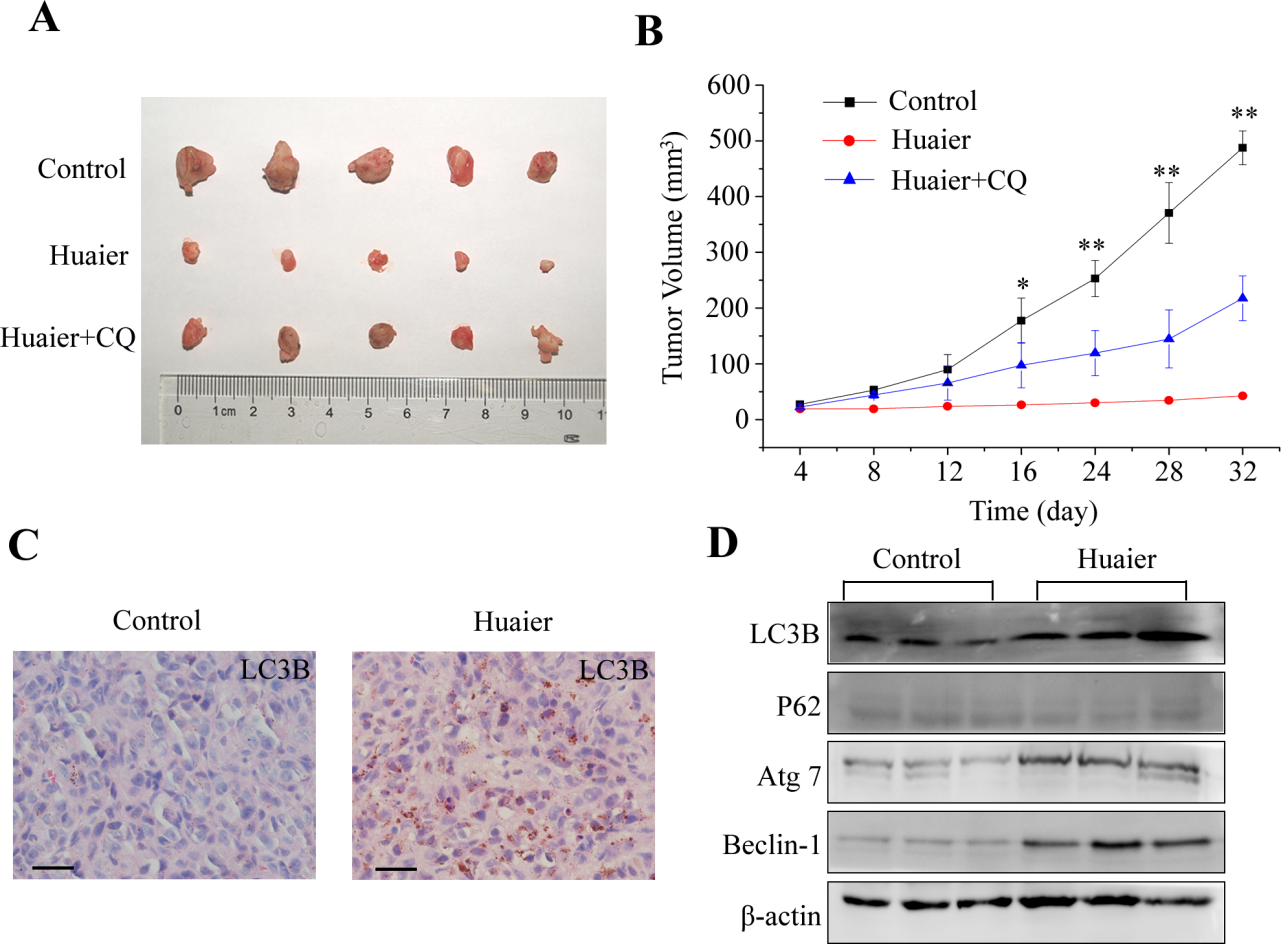
Huaier extract inhibited the growth of breast cancer cells in vivo by inducing autophagy. (A) MDA-MB-231 cells were subcutaneously implanted into the right flank of BALB/c nu/nu mice as described in the materials and methods section. Mice with xenograft tumors were photographed after treatment with PBS (control), Huaier alone or in combination with chloroquine (15 mg/kg once daily) on day 32. (B) The mean volumes of xenograft tumors were measured at 4-day intervals with calipers. Growth curves of xenograft tumors are shown. (C) Representative micrographs of immunohistochemical staining using anti-LC3B antibody. Bars, 30 μm. (D) Western blot analysis of protein expression in tumors taken from mice.

In order to determine whether Huaier extract could inhibit tumor growth in vivo, MDA-MB-231 breast cancer cells were incubated subcutaneously into the right flank of BALB/c nu/nu mice. As shown in Fig 6, Huaier extract treatment inhibited xenograft tumor growth in comparison to the control group. On day 32, the tumor size of the Huaier-treated group significantly diminished from 487.5 ± 30.41 mm^3^ in the control group to 42.5 ± 3.54 mm^3^ in the Huaier-treated group (see Fig 6). When the mice were co-treated with chloroquine and Huaier extract, the inhibitory effect of Huaier extract on tumor growth was partially rescued.

In order to examine the mechanism underlying the inhibition of tumor growth by Huaier extract in vivo, we measured the expression of LC3B using immunohistochemical staining. As shown in Fig 6C, the expression of LC3B in the cytoplasm increased in the tumors treated with Huaier extract compared with the control tumors. Furthermore, Huaier extract increased the expression of LC3B-II, Atg7, Beclin-1 and reduced the expression of p62/SQSTM1 in vivo. These results suggest that Huaier extract inhibited tumor growth in vivo by inducing autophagy.

## Discussion

An increasing body of evidence indicates that natural herbal products may play a promising and potential role in developing novel chemotherapeutics for cancers [31, 32]. In recent years, Huaier extract has been extensively studied for its antitumor effects, including anti-proliferation activity, anti-angiogenesis and anti-metastasis effects via various pathways [11, 33, 34]. Previously, our group reported that Huaier extract induced apoptosis in both ER-positive and ER-negative breast cancer cell lines [9]. In this study, we investigated the role of Huaier extract in inducing autophagy in human breast cancer cells. We demonstrated that Huaier extract triggered autophagic cell death in MDA-MB-231, MDA-MB-468 and MCF7 cells. To date, this is the first report on the capacity of Huaier extract to induce autophagy by the inhibition of the mTOR/S6K pathway.

Autophagy has been observed in response to various stimuli, such as radiation and tamoxifen, thus indicating the potential utility of artificially induced autophagy in cancer therapy [35–37]. During autophagy, portions of the cytoplasm are sequestered into double membrane vesicles known as autophagosomes, which then fuse with lysosomes to form single-membrane autophagolysosomes. The contents of the autophagolysosomes are ultimately degraded by lysosomal hydrolase. Autophagic cells are thus characterized by the accumulation of vacuoles and LC3, which forms the membrane of autophagosome [38, 39]. In this study, we examined autophagy in breast cancer cells induced by Huaier extract through electron microscopy and MDC staining. The results from an analysis of LC3B-Ⅱ expression indicate that the induction of autophagy is dose-dependent (see Fig 2). We further investigated the role of autophagy in Huaier-induced cell death via pretreatment with autophagy inhibitors or the transfection with siLC3B. Our results show that Huaier-induced cell death was rescued by autophagy inhibition (see Figs 3 and 4). Thus, autophagy may play a tumor-suppressive role in breast cancer cells that are treated with Huaier extract.

In recent years, the serine/threonine kinase mTOR has become an important and attractive therapeutic target for cancer therapy and numerous studies demonstrated that mTOR kinase negatively regulates autophagy. mTOR regulates autophagy through two general mechanisms. Firstly, with the help of various downstream effector proteins, mTOR controls transcription and translation [40]. Secondly, it affects the Atg proteins by interfering with the formation of autophagosomes [41]. Our results show that Huaier extract treatment decreases mTOR activity. This inhibitory effect of Huaier extract on mTOR activation was correlated with the loss of phosphorylation of their downstream targets, p70S6K, S6 and 4E-BP1. These findings indicate that Huaier-induced autophagy in breast cancer cells involves an inhibition of the mTOR/S6K signaling pathway.

In summary, we have shown for the first time that Huaier extract induces autophagic cell death in breast cancer cells, both in vitro and in vivo, through the mTOR/S6K pathway. In this study, we provide evidence showing that Huaier extract could act as a new anti-cancer agent for breast cancer by inducing autophagy. Further studies are required to explore and identify the responsible components of Huaier extract and their molecular mechanisms.
